# Prevalence of Gastrointestinal Helminths of Dogs and Associated Factors in Hawassa City of Sidama Region, Ethiopia

**DOI:** 10.1155/2023/6155741

**Published:** 2023-02-06

**Authors:** Teshager Dubie, Shilmat Sire, Gizachew Fentahun, Fanuel Bizuayehu

**Affiliations:** College of Veterinary Medicine, Samara University, P.O. Box 132, Semera, Ethiopia

## Abstract

A cross-sectional study was carried out in the Sidama region's Hawassa town from March 2021 to July 2021 with the aim of estimating the prevalence and associated factors of dog gastrointestinal helminths. A total of 384 dogs were randomly selected, and their feces were examined using a flotation technique. For data analysis, descriptive statistics and chi-square analyses were employed, and a *P*-value of less than 0.05 was considered as significant. Accordingly, 56% (*n* = 215; 95% CI, 49.26–62.66) of dogs had gastrointestinal helminth parasite infection, of which 42.2% (*n* = 162) had a single infection and 13.8% (*n* = 53) had a mixed infection. In this study, *Strongyloides* sp. was the most detected helminth (24.2%), followed by *Ancylostoma* sp. (15.37%), *Trichuris vulpis* (14.6%), *Toxocara canis* (5.73%), *Echinococcus* sp. (5.47%), and *Dipylidium caninum* (4.43%). Out of the total sampled dogs tested positive for one or more gastrointestinal helminths, 37.5% (*n* = 144) were males and 18.5% (*n* = 71) were females. The total prevalence of helminth infections did not change significantly (*P* > 0.05) by the gender, age, and breed of dogs. The present study's high prevalence of dog helminthiasis reflects a high occurrence of infection and a concern to the public's health. In light of this conclusion, it is advised that dog owners improve their standards of hygiene. In addition, they should regularly take their animals to veterinary care and frequently administer the appropriate anthelmintics available to their dogs.

## 1. Introduction

The dog was the first domesticated animal and has coexisted with humans for a very long time [1]. The advantage of domestication of dog was important in hunter-gatherer societies as hunting allies, bodyguards against predators, a social function, and giving companionship. On the other hand, one of the disadvantages of dogs' domestication is the possibility of transmission of zoonotic parasites, especially helminths. The most common zoonotic helminths of dogs are *Strongyloides stercoralis*, *Ancylostoma caninum*, *Dipylidium caninum*, *Toxocara canis*, and *Echinococcus granulosus*, which means they can be infectious for humans and cause a variety of diseases like hydatidosis, visceral larva migrans, and cutaneous larva migrans [2]. As dogs and humans coexist in close quarters, contamination of human food, water, or hands by these gastrointestinal (GI) helminths' infectious stages can result in life-threatening infections. Particularly in developing nations, where control measures are frequently not used [5–7].

Dogs of all ages are susceptible to these parasites, which manifest a variety of symptoms according to the host's immunity level, parasite species, and density (parasitic load). Clinical symptoms of intestinal parasites in dogs include vomiting, diarrhea, anemia, anorexia, underweight, dermatitis, dehydration, and deterioration of health [5]. These signs are attributed to intestinal obstruction, irritation, maldigestion, malabsorption, and protein losing gastroenteropathy induced by the parasites [6] and resulting in lowered resistance to disease, retarded growth, and reduce work efficiency [7]. Even though there are effective treatments available to treat parasites, the majority of parasites in dogs have highly complicated life cycles that make treatment hard.

In Ethiopia, many families keep one or more dogs either as hunting or guard dogs, and a large number of dogs are commonly seen at abattoirs, butcher shops, market place, and streets. Due to their closest contact with man [8, 9], GI helminths of dogs may be a threat to human health [10, 11]. The majority of dogs in Ethiopia are allowed to wander, and it has been found that these canines have the highest parasite burdens. In the absence of widespread public awareness of these concerns, the existence of a significant number of strays and unconfined canines with a high frequency of zoonotic parasites that contaminate the environment is extremely worrisome. Based on the helminth genus involved, the animal species, and local environmental factors including humidity, temperature, rainfall, vegetation, and management practices, it has been demonstrated that the prevalence of helminth parasites varies significantly from one geographic region to another [8, 12]. Therefore, in order to successfully develop and implement helminth parasites control strategy in the dog population, periodic estimation of the incidence of these parasites within a given region is required. The frequency of numerous GI parasites in dogs has received little investigation and less attention in Ethiopia, despite the fact [13, 14].

In Ethiopia generally and in the study area specifically, extremely less attention was given to the incidence of the many GI parasites that affect dogs. There is also a dearth of research on helminth parasites [13, 14]. Hence, the objective of this study was to estimate the prevalence of GI parasites in dogs and associated factors in Hawassa city, Sidama region, Ethiopia.

## 2. Materials and Methods

### 2.1. Description of the Study Area

The study was conducted in Hawassa town from March 2021 to July 2021 in Hawassa, capital city of the Sidama Regional State, South Ethiopia. Hawassa lies between 4°27′ and 8°30′ N latitude, and 34°21′ and 39°1′ E longitude at an altitude of 1790 m above sea level (m.a.s.l). The area receives 800–1000 mm average annual rainfall of which 67% falls in the long rainy season, which extends from June to September. The total human population of Hawassa is estimated to be 150,000. Hawassa town covers an area of 50 km^2^. The mean minimum and maximum temperature of the area are 20.1 and 30°C, respectively, and means relative humidity is 51.8% [15].

### 2.2. Study Design and Study Population

A cross-sectional study was carried out to estimate the prevalence and to determine the frequency of GI helminth of dog in Hawassa city using a flotation technique and analyzed variables. The study populations were selected by simple random sampling method. The study populations were domesticated local and exotic breeds of dogs that live in and around Hawassa town. Both sexes and all age groups of dogs in Hawassa city were included in the study. For simplicity, dogs up to 1 year of age were grouped as young while those older than 1 year as adult dogs.

### 2.3. Sampling Technique and Sample Collection

Simple random sampling technique was used to determine the abundance of intestinal helminth of dog in Hawassa town. Fecal samples were collected from 384 dogs brought to Hawassa town veterinary clinic and dogs kept at home in the town. All samples were collected from four government veterinary clinics and home to home researching of domesticated dogs. Before sample collection, the researchers checked that the study populations were not dewormed to be included in the study population (informed by dogs' owners). The samples were collected directly from the rectum of the dogs and from top layers of fresh voided feces and kept into labeled disposable container and transported immediately to Hawassa University Veterinary Parasitology laboratory [16]. The history and sex of dogs were recorded during examination, and approximate age of dogs was estimated using criteria described by Pace and Wakeman [17]. Those dogs less than 1 year were classified as young (*n* = 166) and those over 1 year as adult (*n* = 218).

### 2.4. Sample Size Determination

To calculate the total sample size, the following parameters were used: 95% level of confidence (CL), 5% desired level of precision, and with the assumption of 50% expected prevalence of helminths in dogs in the study area. The sample size was determined using the formula given by [18];

(1) $$\begin{array}{c} n=\frac{Z^{2}\times P_{\exp}\left(1-P_{\exp}\right)}{d^{2}}, \end{array}$$

where *n* = required sample size, *P*_exp_ = expected prevalence, *d* = absolute precision, *n* = required sample size, *P*_exp_ = expected prevalence, *d* = absolute precision, *Z* = multiplier from normal distribution at 95% confidence interval (1.96). Accordingly, 384 fecal samples were collected from the study population.

### 2.5. Study Methodology

The samples were processed using a simple flotation technique briefly as follows. The eggs were identified based on the general characteristics described by Hendrix [19] and Soulsby [20]. Approximately 3 g of feces was transferred to a 50 mL centrifuge tube. It was then half-filled in phosphate buffered saline (1× PBS) and vortexed. The suspension was mixed and centrifuged at 3000 rpm for 10 minutes. After centrifugation, the supernatant was poured off and the pellet (~5 mL) retained. The pellet was re-suspended in distilled water to 50 mL, mixed, centrifuged, and the supernatant poured off as above. Then saturated sodium chloride solution (specific gravity 1.20) was added to 30 mL mark, briefly mixed, and carefully topped up with distilled water until 50 mL mark by letting the water run down the wall of the tube, so that two phases were created. The mixture was centrifuged at 3000 rpm for 10 minutes, and the water phase in the upper most layers and the interface was transferred to the new tube using a plastic pipette. Following that, the new tube was topped up using distilled water, mixed, centrifuged as above, and the supernatant poured off retaining 5 mL pellet. The last washing step was repeated twice more to remove any remnant salt and have a clean sample for analysis. Finally, the bottom 5 mL pellet was collected, and tests are performed [21].

### 2.6. Data Management and Analysis

The coprological examination of raw data that were recorded, edited, and analyzed from this study were entered into Microsoft Excel database system, and computation of descriptive statistics was conducted using SPSS version 16.0. Descriptive statistics such as percentages, proportions, and frequency distributions were applied to compute some of the data. Chi-square (X^2^) was used to check association between the prevalence of helminth parasite infection and associated factors. In all the analyses, 95% confidence interval was considered, and the *P* < 0.05 was used for statistical significance level between the associated factors and parasitological positivity.

## 3. Results

In this study, out of 384 fecal samples tested using coproscopical examination, an overall prevalence of 56% (*n* = 215) helminth infection was found to be positive for various species of GI helminths. Out of 215 positive samples, 42.2% (*n* = 162) was single helminth infection and 13.8% (*n* = 53) was mixed infection as depicted (Table 1). The single helminth infection consists of *S. stercoralis*, *Trichuris vulpis*, *Ancylostoma caninum*, *E. granulosus*, *Dipylidium caninum*, *T. canis,* and double infection entails of *Ancylostoma* and *Strogyloides* sp.*, Strongyloides* and *Toxocara* sp., *Echinococcus* and *Strongyloides* sp., *Dipylidium* and *Trichuris* sp., *Toxocara* and *Ancylostoma* sp., and *Strongyloides* and *Trichuris* sp. of helminths.

Based on the characteristics of their eggs, six GI parasite species in dogs were identified in this study. The most frequent helminth parasite species identified was *S. stercoralis* (24.2%), whereas *D. caninum* (4.43%) was the least prevalent as indicated in (Table 2).

**Figure 1:**
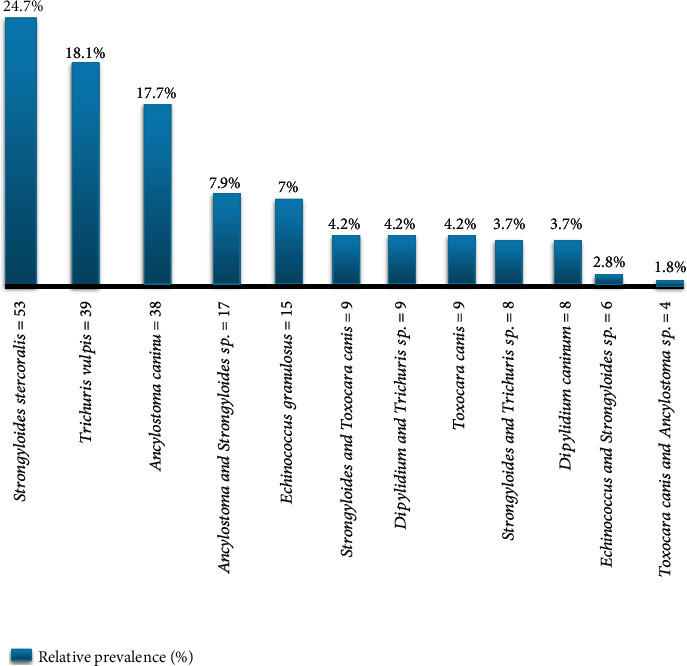
Genus of gastrointestinal helminths and relative prevalence in dogs at Hawassa town, Ethiopia.

As the current study finding revealed that the study populations were infected with both single helminth infections and double helminth infections with different relative prevalence magnitude as indicated in Figure 1.

Among the variables analyzed, no significant difference was observed between sexes, age groups, and breed in terms of parasitological positivity (*P* > 0.05) (Table 3).

## 4. Discussion

The overall prevalence of GI parasites of dogs from Hawassa was 56% (*n* = 215). Many dogs were infected by at least one species of helminth parasites. In this study, the identified species were *T. canis*, *Echinococcus* sp., *Strongyloides* sp., *D. caninum*, *T. vulpis*, and *Ancylostoma* sp. Studies in Ethiopia and other nations found similar prevalence survey results of GI parasites in dogs by Endrias et al. [22] in Ambo town, Central Ethiopia (52.86%), 51% in Debre Zeit, Ethiopia [13], and Panigrahi et al. [23] reported (41.56%) in India. In Ethiopia, previous copro-parasitological studies with other canine populations indicated higher prevalence than the current study result in the cities of Bahir Dar (75.26%) by Abere et al. [24], Hawassa (84.6%) by Dejene et al. [25], Adama town, Ethiopia (82.02%) by Merga and Sibhat [26], Mekele city (73.3%), Ethiopia by Gugsa et al. [27], and Hawassa town (89.3%) by Mekbib et al. [28]. The other possible justification of higher frequency of these parasites could indicate the high soil contamination by infective nematode larvae. On the other hand, this study finding is slightly higher than previous study findings of Guesh et al. [29] in Mekelle city, Ethiopia who reported a prevalence of 30.5%, Teresa et al. [30] in Portuguese city of Ponte de Lima with a report of 14.25%. The wide range of variations in the prevalence of the GI parasites was attributed to geographical location, the presence or absence of intermediate hosts of the correlating parasites, the status of animal ownership, sampling protocols, demographic factors, the use of anthelmintics, and diagnostic methods [12]. In particular, the availability of abattoirs during the study period, lack of an adequate waste disposal system, the community's high trends of raw meat consumption, sample collection from clinical case suspected dogs, and poor sanitation practices of dog owners may all contribute to an overestimation of the prevalence of this study [31].

The prevalence of multiple GI helminth species in a single host was 13.8% (*n* = 53). It might be as a result of the dogs acting as scavengers and not receiving routine veterinarian care. On the other hand, 42.4% (*n* = 162) of the dogs had at least one helminth infection. In this study, the most common parasite infecting dogs in Hawassa was *Strongyloides* sp., followed by *Ancylostoma* sp. Moreover, the co-infection by these two nematodes was the mixed infection most detected. The other hypothesis for the higher frequency of these parasites could suggest a relation to soil nematode larvae infestation. The present study finding showed a higher prevalence of *S. stercoralis* than the previous reports by Yacob et al. [13] and Eleni et al. [32], who reported 14.29% and 4.29% in Ambo and Gondar town, respectively. In this study, *A. caninum* (15.36%) was the second most prevalent parasite infecting dogs in Hawassa. This study is supported by previous reports of Endrias et al. [22] in Debre-Zeit and Yacob et al. [13], which the predominant parasite species reported in these studies was *S. stercoralis* followed by *Ancylostoma* species. The current study found no significant difference (*P* > 0.05) between males and females in the occurrence of intestinal helminths in dogs. The current study result is in line with previous studies [13, 33]. *Dipylidium caninum* was found with the least prevalent (4.427%). This finding is comparable with the reported in Tabriz Iran (7%) by Garedagh and Mashsaci [34] and in Brazil (2.4%) by Katagiri and Oliveira-Sequeira [35]. But higher values were recorded (25.8%) by Degefu et al. [36] in Jimma, Ethiopia, (25.7%) Endrias et al. [22] in Ambo town, Ethiopia, and (75%) reported by Umar [37] in Nigeria. The intensity of flea infestation in dogs in the study area, geographic regions, the status of environmental sanitation, poor body condition, and degree of dog's home confinement are all plausible reasons for the variations in the prevalence of *D. caninum* infection in dogs across different nations [38, 39].

- More than half of dog owners either allow their animals to urinate outdoors or dispose of their pets' waste there. The majority of dog owners clean dog waste without using protective gear such as gloves, faces masks, boots, coveralls, or gowns, placing them at high risk of contracting serious zoonotic parasite diseases like *S. stercoralis, A. caninum, D. caninum, T. canis,* and *E. granulosus*. Adopting and implementing appropriate preventive measures from One Health approach point of view such as public awareness creation, regular deworming, and keeping dogs housed and properly fed may be important strategies to avoid transmission of these zoonotic helminths. Dog owners can either give their dogs raw, condemned offal from butcher shops or backyard slaughter, or they can let the animals hunt for food on their own, which may include contaminated raw offal. By being a source of infection and spreading numerous parasite zoonotic diseases, the practice of feeding dogs uninspected, inadequately prepared, or uncooked offal may raise the danger of public exposure. In this study finding, *Toxocara* species and *E. granulosus* may be detected due to this practice.
- Limitations of the present study included sampling only the wet season, search for veterinary care by owners who identified some clinical change in their animals that might have had an effect on the final prevalence estimation, prevalence cannot be extrapolated to the general canine population of Hawassa as stray dogs were not included.

## 5. Conclusions

The present study's high incidence of canine helminthiasis indicates a high rate of infection with *S. stercoralis*, *A. caninum,* and *T. vulpis* species are more prevalent parasites. It is recommended that dog owners should improve their hygiene standards and regularly take their dogs to veterinary care. Moreover, awareness should be created on the prevention and control methods of helminthiasis, especially the zoonotic ones. Due to the narrow focus of this study, primarily GI helminths and only the dry season of the year, detailed epidemiological studies should be further carried out.

## Figures and Tables

**Table 1:** Distribution of mono and polyparasitised dogs with gastrointestinal helminths according to sex, age group, and breed in Hawassa city, Ethiopia.

Variables	Category	No. animal examined	Positive sample	Prevalence (%)	Double infection	Prevalence (%)	Single infection	Prevalence (%)
Sex	Male	245	144	37.5	40	10.4	104	27.1
	Female	139	71	18.5	13	3.4	58	15.1
Age	Young	166	100	26	20	5.2	80	20.8
	Adult	218	115	30	33	8.6	82	21.4
Breed	Local	143	77	20.1	20	5.2	57	14.8
	Exotic	241	138	35.9	33	8.6	105	27.4
Total		384	215	56	53	13.8	162	42.2

**Table 2:** Prevalence of various gastrointestinal parasite species in dogs at Hawassa town, Ethiopia.

Species	Positive samples	Percentage
*Toxocara canis*	22	5.73%
*Echinoccocus* sp.	21	5.47%
*Ancylostoma* sp.	59	15.37%
*Dipylidium caninum*	17	4.43%
*Trichuris vulpis*	56	14.58%
*Strongyloides stercoralis*	93	24.2%

**Table 3:** Analyzed variables associated with helminth infection of dogs in the Hawassa city of Sidama region, Ethiopia.

No.	Associated risk factors		Positive	Percentage	*X* ^2^	*P*-value
1	Sex	Males	144	67.0%	2.1	0.17
		Females	71	33.0%		
2	Age	Young (up 1 year)	100	46.5%	2.13	0.15
		Adult (above years)	115	53.5%		
3	Breed	Local	77	35.8%	0.43	0.52
		Exotic	138	64.2%		

## Data Availability

The data sets used and/or analyzed during the current study available from the corresponding author on reasonable request.
